# Genetic Variation of *Sclerotinia sclerotiorum* from Multiple Crops in the North Central United States

**DOI:** 10.1371/journal.pone.0139188

**Published:** 2015-09-29

**Authors:** Laura Aldrich-Wolfe, Steven Travers, Berlin D. Nelson

**Affiliations:** 1 Biology Department, Concordia College, Moorhead, Minnesota, United States of America; 2 Department of Biological Sciences, North Dakota State University, Fargo, North Dakota, United States of America; 3 Department of Plant Pathology, North Dakota State University, Fargo, North Dakota, United States of America; Chinese Academy of Sciences, CHINA

## Abstract

*Sclerotinia sclerotiorum* is an important pathogen of numerous crops in the North Central region of the United States. The objective of this study was to examine the genetic diversity of 145 isolates of the pathogen from multiple hosts in the region. Mycelial compatibility groups (MCG) and microsatellite haplotypes were determined and analyzed for standard estimates of population genetic diversity and the importance of host and distance for genetic variation was examined. MCG tests indicated there were 49 different MCGs in the population and 52 unique microsatellite haplotypes were identified. There was an association between MCG and haplotype such that isolates belonging to the same MCG either shared identical haplotypes or differed at no more than 2 of the 12 polymorphic loci. For the majority of isolates, there was a one-to-one correspondence between MCG and haplotype. Eleven MCGs shared haplotypes. A single haplotype was found to be prevalent throughout the region. The majority of genetic variation in the isolate collection was found within rather than among host crops, suggesting little genetic divergence of *S*. *sclerotiorum* among hosts. There was only weak evidence of isolation by distance. Pairwise population comparisons among isolates from canola, dry bean, soybean and sunflower suggested that gene flow between host-populations is more common for some crops than others. Analysis of linkage disequilibrium in the isolates from the four major crops indicated primarily clonal reproduction, but also evidence of genetic recombination for isolates from canola and sunflower. Accordingly, genetic diversity was highest for populations from canola and sunflower. Distribution of microsatellite haplotypes across the study region strongly suggest that specific haplotypes of *S*. *sclerotiorum* are often found on multiple crops, movement of individual haplotypes among crops is common and host identity is not a barrier to gene flow for *S*. *sclerotiorum* in the north central United States.

## Introduction

The pathogen *Sclerotinia sclerotiorum* (Lib.) de Bary has been the focus of research since it was first described over a hundred years ago in part because this fungus is capable of infecting many different crops [1]. Over 400 different plant species can be infected by this pathogen [2]. While the effects of *S*. *sclerotiorum* vary among host plant species, it is capable of causing severe reductions in crop yield and significant economic impacts [1]. Understanding the population structure and genetic diversity of *S*. *sclerotiorum* may provide insight into modes of reproduction, spread of the pathogen and severity of disease on crops.

*Sclerotinia sclerotiorum* is capable of reproducing both asexually and sexually. Sexual reproduction occurs through carpogenic germination of sclerotia resulting in apothecia then ascospore production. However since the fungus is homothallic, a single ascospore can complete the life cycle. Asexual reproduction occurs by myceliogenic germination of sclerotia directly resulting in new sclerotia or mycelium that eventually produces sclerotia. Therefore, both sexual and asexual reproduction results in a primarily clonal population structure. Asexual reproduction through the production of sclerotia is considered most common [3, 4, 5, 6]. However, there is some evidence of recombination through sexual reproduction in populations [7, 8, 9, 10], which may increase the genetic diversity and adaptability of the pathogen. Modes of reproduction are likely to affect patterns of genetic diversity. In addition to promoting genetic recombination, sexual reproduction gives rise to wind dispersal. Asexual reproduction is more likely to lead to short distance dispersal. The fact that *S*. *sclerotiorum* can infect a wide array of plant species suggests that there are few genetic constraints on its propagation.

Clonal lineages of *S*. *sclerotiorum* have been distinguished from each other by the identification of mycelial compatibility groups (MCGs), which are determined via an assay of phenotypes for a self-recognition system controlled by multiple loci [8]. In addition, DNA profiles using a variety of different methods such as microsatellites or the nuclear ribosomal RNA gene, have also commonly been used to characterize genetic diversity in populations of *S*. *sclerotiorum* [3, 11, 12, 13, 14, 15, 16]. Most studies of *S*. *sclerotiorum* examining both MCGs and DNA profiles have shown that they are closely correlated [3, 5, 11, 13, 14, 17], but Atallah et al. [7] and Malvarez et al. [15] found little relationship between MCGs and neutral genetic markers indicating the stability of this relationship may differ between populations or depend on markers used. There have been numerous studies of genetic diversity in populations of *S*. *sclerotiorum*, but few have studied populations over multiple crops with wide geographic distribution [9, 13, 15, 18].

The North Central region of the United States has the greatest acreage of field crops susceptible to *S*. *sclerotiorum*. In the twelve north central states there are over 27 million hectares of susceptible field crops (canola, *Brassica napus* L.; dry bean, *Phaseolus vulgaris* L.; soybean, *Glycine max* (L.) Merr.; sunflower, *Helianthus annuus* L.) in which this pathogen can reproduce. The majority of those hectares (90%) are soybean, which spans the entire breadth, both north and south, and east and west, of the region. *Sclerotinia sclerotiorum* is a common pathogen among these crops and causes substantial losses [1]. To date, there has been no study on genetic diversity of this important pathogen over the entire region that has included isolates of the pathogen from multiple crops.

The objectives of this research were to use genetic markers based on simple sequence repeats (microsatellites) to estimate levels of genetic diversity in populations of *S*. *sclerotiorum*, assess the correspondence between microsatellite haplotype and MCG, and use patterns of microsatellite haplotype distribution to test for divergence of this pathogen among the different host crops in the North Central region of the United States. Our study was designed to address the following questions: 1) What is the genetic diversity of *S*. *sclerotiorum* in the North Central region of the United States? 2) Do MCGs correspond to microsatellite haplotype among *S*. *sclerotiorum* isolates? 3) Have populations of *S*. *sclerotiorum* on different host crops diverged genetically from one another? and 4) Is there evidence for geographic differences in the frequency of different haplotypes?

## Materials and Methods

### Isolates

**Fig 1:**
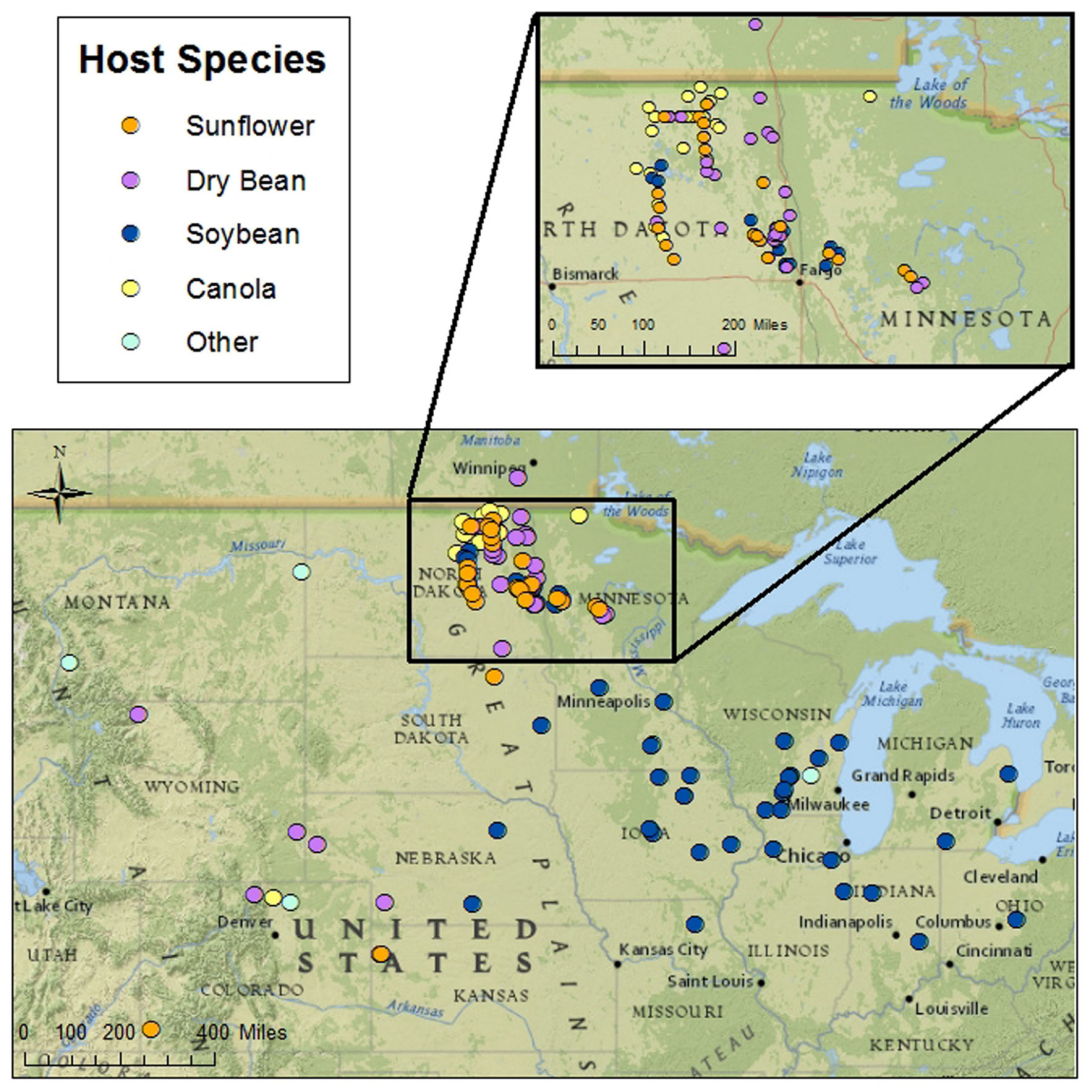
Geographic distribution of isolates of *Sclerotinia sclerotiorum* from the North Central United States. There were 145 isolates from the north central area and several adjacent western states, and Manitoba, Canada. The map was produced with Ersi software ArcGIS 10.2 using a National Geographic basemap of North America.

Sclerotia of *S*. *sclerotiorum* were collected by the authors from various crops in 73 commercial crop fields in eastern North Dakota and northwestern Minnesota during fall 2008. An additional 72 collections of sclerotia were obtained from other researchers in 12 states in the north central United States, three western states and Manitoba, Canada, that border the north central region, for a total of 145 isolates (Table 1; Fig 1). Most of the sclerotia obtained by other researchers were collected between 2000 and 2008, but there were 5 samples that were collected between 1987 and 1998. Those researchers collected the samples within their respective states while examining Sclerotinia diseases on crops and weeds (listed in Table 1). No permits or special permissions were required by the authors or the other researchers within their respective states to collect diseased plant materials. The sites sampled were all commercial crop fields or specific university crop research sites. None of the samples in this study were collected from endangered or protected plant species. For many of the samples obtained from other researchers, the precise GPS coordinates of sample locations were not available, thus an approximate GPS coordinate was obtained to prepare the map in Fig 1. Sclerotia from other researchers were received by the authors between November 2008 and January 2010 using a USDA Animal and Plant Health Inspection Service (APHIS) permit to move live plant pests. APHIS permit P526P-08-02796 was issued to B. Nelson in September 2008 with expiration in September 2011. A single sclerotium from each collection was surface-disinfected for 30 s in 0.5% NaOCl, transferred to potato dextrose agar (PDA) and incubated at 23° C in the dark. Once a sclerotium had germinated, hyphal tips were transferred to fresh PDA and the resulting sclerotia were collected and stored at -20 or 4°C. Cultures used for experiments were always initiated from sclerotia from storage.

**Table 1:** Correspondence between mycelial compatibility group (MCG) and haplotype at twelve polymorphic microsatellite loci for 145 isolates of *Sclerotinia sclerotiorum* collected from nine host species in the North Central United States.

MCG	Haplotype	Year	State/Province	Host	Host common name	Number of isolates	Isolate designations
1	1	2007	North Dakota	*Brassica napus*	canola	1	100
2	2a	2008	Minnesota	*Phaseolus vulgaris*	dry bean	1	137
2	2a	2008	Nebraska	*Glycine max*	soybean	1	191
2	2a	2008	North Dakota	*Brassica napus*	canola	2	175,181
2	2a	2008	North Dakota	*Phaseolus vulgaris*	dry bean	2	101,122
2	2a	2008	North Dakota	*Glycine max*	soybean	1	132
2	2a	2008	South Dakota	*Glycine max*	soybean	1	201
2	2a	2007	North Dakota	*Brassica napus*	canola	1	800
2	2a	1996	Colorado	*Phaseolus vulgaris*	dry bean	1	196
2	2b	2008	Manitoba, Canada	*Phaseolus vulgaris*	dry bean	1	103
3	3a	2008	North Dakota	*Brassica napus*	canola	1	159
3	3a	2008	North Dakota	*Phaseolus vulgaris*	dry bean	1	102
3	3a	2007	North Dakota	*Brassica napus*	canola	2	700,900
3	3a	—	Minnesota	*Daucus carota*	carrot	1	205
3	3a	2008	North Dakota	*Helianthus annuus*	soybean	4	129,134,180,189
3	3b	2008	North Dakota	*Phaseolus vulgaris*	dry bean	1	156
3	3b	2008	North Dakota	*Glycine max*	soybean	1	240
3	3b	2008	North Dakota	*Helianthus annuus*	sunflower	1	188
4	4	2008	North Dakota	*Phaseolus vulgaris*	dry bean	1	104
5	2a	2008	North Dakota	*Phaseolus vulgaris*	dry bean	1	105
5	2a	2008	North Dakota	*Glycine max*	soybean	1	116
6	6a	2008	Minnesota	*Helianthus annuus*	sunflower	2	136,139
6	6a	2008	North Dakota	*Phaseolus vulgaris*	dry bean	1	106
6	6b	2008	North Dakota	*Glycine max*	soybean	1	176
7	7	2008	North Dakota	*Glycine max*	soybean	1	107
8	8a	2008	Iowa	*Glycine max*	soybean	1	213
8	8a	2008	North Dakota	*Brassica napus*	canola	2	167,186
8	8a	2008	North Dakota	*Phaseolus vulgaris*	dry bean	4	109,115,119,133
8	8b	2008	North Dakota	*Phaseolus vulgaris*	dry bean	1	120
8	8b	2007	Montana	*Carthamus tinctorius*	safflower	1	244
8	8b	2006	Minnesota	*Glycine max*	soybean	1	203
8	8c	2008	North Dakota	*Glycine max*	soybean	1	239
8	8c	2008	North Dakota	*Helianthus annuus*	sunflower	1	183
9	9	2008	Illinois	*Glycine max*	soybean	1	148
9	9	2008	Indiana	*Glycine max*	soybean	2	206,207
9	9	2008	Iowa	*Glycine max*	soybean	6	211,212,214215,216,217
9	9	2008	Minnesota	*Glycine max*	soybean	4	145,146,197,204
9	9	2008	Minnesota	*Helianthus annuus*	sunflower	1	141
9	9	2008	Nebraska	*Glycine max*	soybean	1	153
9	9	2008	North Dakota	*Brassica napus*	canola	1	150
9	9	2008	North Dakota	*Phaseolus vulgaris*	dry bean	7	108,117,124,131151,157,184
9	9	2008	North Dakota	*Glycine max*	soybean	5	113,114,125,135,178
9	9	2008	North Dakota	*Helianthus annuus*	sunflower	2	126,128
9	9	2007	Illinois	*Glycine max*	soybean	1	147
9	9	2007	North Dakota	*Brassica napus*	canola	1	110
9	9	2004	Missouri	*Glycine max*	soybean	1	234
9	9	2004	Wisconsin	*Nicotiana tabacum*	tobacco	1	233
9	9	2003	Wisconsin	*Nicotiana tabacum*	tobacco	1	232
9	9	2002	Ohio	*Glycine max*	soybean	1	210
9	9	2002	Wisconsin	*Glycine max*	soybean	1	226
9	9	2000	Wisconsin	*Glycine max*	soybean	4	220,221,223,224
11	11	1987	Colorado	*Solanum tuberosum*	potato	1	198
12	12a	2008	Minnesota	*Glycine max*	soybean	1	140
12	12a	2008	North Dakota	*Helianthus annuus*	sunflower	1	123
12	12b	2008	North Dakota	*Phaseolus vulgaris*	dry bean	1	118
12	12b	2008	North Dakota	*Glycine max*	soybean	1	127
12	12b	2006	Colorado	*Helianthus annuus*	sunflower	1	193
13	13	2008	North Dakota	*Brassica napus*	canola	1	168
14	14	2007	North Dakota	*Brassica napus*	canola	1	111
15	15	2008	Minnesota	*Brassica napus*	canola	1	190
15	15	2008	North Dakota	*Brassica napus*	canola	2	171,177
15	15	2007	North Dakota	*Brassica napus*	canola	1	112
15	15	1992	Colorado	*Brassica napus*	canola	1	192
16	16	2008	North Dakota	*Glycine max*	soybean	1	130
17	17	2008	North Dakota	*Brassica napus*	canola	1	185
18	18	2008	Minnesota	*Phaseolus vulgaris*	dry bean	1	138
19	19	2008	Minnesota	*Glycine max*	soybean	1	142
19	19	2003	Wisconsin	*Glycine max*	soybean	1	229
20	20	2008	Minnesota	*Helianthus annuus*	sunflower	1	143
21	21	2008	Minnesota	*Glycine max*	soybean	1	144
23	23	2008	North Dakota	*Helianthus annuus*	sunflower	1	149
24	24	2008	Nebraska	*Phaseolus vulgaris*	dry bean	1	154
25	25a	2008	Kansas	*Helianthus annuus*	sunflower	1	246
25	25a	2008	North Dakota	*Phaseolus vulgaris*	dry bean	1	121
25	25a	2008	North Dakota	*Helianthus annuus*	sunflower	1	155
25	25b	2008	North Dakota	*Glycine max*	soybean	1	241
28	28a	2008	North Dakota	*Phaseolus vulgaris*	dry bean	1	158
28	28a	2008	North Dakota	*Helianthus annuus*	sunflower	2	160,161
28	28a	2007	North Dakota	*Brassica napus*	canola	1	500
28	28b	2008	North Dakota	*Helianthus annuus*	sunflower	1	166
30	30	2008	North Dakota	*Helianthus annuus*	sunflower	1	163
31	31	2008	North Dakota	*Brassica napus*	canola	1	165
33	33	2008	North Dakota	*Phaseolus vulgaris*	dry bean	1	169
34	34	2008	North Dakota	*Brassica napus*	canola	1	170
36	36	2008	North Dakota	*Phaseolus vulgaris*	dry bean	1	172
37	37	2008	North Dakota	*Brassica napus*	canola	1	173
38	38	2008	North Dakota	*Helianthus annuus*	sunflower	2	174,182
43	43	2008	North Dakota	*Glycine max*	soybean	1	179
46	46	2008	North Dakota	*Helianthus anuus*	sunflower	1	187
**49**	12a	2008	Nebraska	*Phaseolus vulgaris*	dry bean	1	195
**52**	23	2008	Wyoming	*Phaseolus vulgaris*	dry bean	1	200
**53**	53	2008	South Dakota	*Helianthus annuus*	sunflower	1	202
56	12a	1997	Iowa	*Glycine max*	soybean	1	208
57	57	1998	Illinois	*Glycine max*	soybean	1	209
60	60	2008	Michigan	*Glycine max*	soybean	2	218,219
61	61	2002	North Dakota	*Brassica napus*	canola	1	230
61	61	2000	Wisconsin	*Glycine max*	soybean	1	222
62	62	2003	Wisconsin	*Glycine max*	soybean	2	227,228
63	24	2008	Wyoming	*Phaseolus vulgaris*	dry bean	1	199
64	64	2002	Wisconsin	*Glycine max*	soybean	1	225
66	66	2002	North Dakota	*Brassica napus*	canola	1	231
69	69	2005	Montana	*Cynoglossum officinale*	houndstonque	1	243
71	71	2008	Kansas	*Phaseolus vulgaris*	dry bean	1	247
73	73	2007	North Dakota	*Brassica napus*	canola	1	600
77	77	2008	North Dakota	*Brassica napus*	canola	1	162
78	19	2008	North Dakota	*Brassica napus*	canola	1	164

### Mycelial Compatibility Groups

MCG tests were completed for all of the *S*. *sclerotiorum* isolates sampled. Mycelial plugs 6 mm in diameter were obtained from the edge of 72 hr old colonies growing on PDA at 23°C in the dark. Pairings were conducted by placing plugs of the test isolates on opposite sides of 100 x 15 mm petri dishes on PDA amended with McCormick’s red food color (100 μl/L of medium) [19]. Cultures were then incubated in the dark at 23°C for 3 to 4 d. Compatible isolates were distinguished by intermingling hyphae, the absence of accumulated red dye, and identical appearance to self-self pairings of individual isolates [19]. Incompatible isolates produced a barrage zone, consisting of a region of sparse mycelia accompanied by an obvious red line of accumulated dye. Some pairings required up to 7 d of growth to determine compatibility. Groups of 10 to 20 isolates were grown in all possible pairwise combinations to determine initial MCGs. Subsequently, isolates were compared across groups to consolidate MCGs. If there was more than one isolate in an MCG, then tests across MCGs were repeated with different isolates.

### DNA isolation

Mycelial plugs were obtained from the edge of 3–4 day-old colonies of *S*. *sclerotiorum* growing on PDA and transferred to sterile Whatman polycarbonate membrane filters (0.4 μm pore size) on PDA. Cultures were incubated in the dark at 23°C for 2 to 3 d. Mycelium was scraped from the filter, lyophilized and stored at -80°C. Genomic DNA was extracted with the DNeasy Mini Plant Kit (Qiagen, Valencia, California) according to the manufacturer’s protocol, with the following modifications. Cells were disrupted by shaking on a vortex adaptor (MO BIO Laboratories, Carlsbad, California) at maximum speed for 5 min with 5 to 10, 2 mm–diameter, zirconia beads (BioSpec Products, Bartlesville, Oklahoma). DNA extracts were diluted to 20 ng μl^-1^ in ultrapure water and stored at -80°C.

### Microsatellite loci

Twelve microsatellite primer pairs (Table 2) developed by Sirjusingh and Kohn [20] were used to characterize 145 isolates (a thirteenth locus, 42–4, was found to be monomorphic for this collection of isolates) in a 3-primer system with a fluorescently-labeled universal primer. Each forward primer included a universal M13 tail 5’-CAGTCGGGCGTCATCA-3’ at the 5’ end (technique described in Boutin-Ganache et al. [21] and modified by Travis Glenn [http://dna.uga.edu/protocols/capillary-genotyping/]). Each PCR was conducted in a volume of 20 μl with 1× PCR buffer, 2 mM MgCl_2_, 0.2 mM dNTPs, 0.025 μM forward primer, 0.25 μM reverse primer, 0.25 μM universal M13 primer 5’-CAGTCGGGCGTCATCA-3’ labeled with PET, VIC, or 6FAM fluorophores (Applied Biosystems, Foster City, California), 1 U Platinum^®^ Taq DNA polymerase (Invitrogen, Carlsbad, California), and 40 ng genomic DNA. A single fluorophore was assigned to each locus to avoid errors in allele size estimate caused by differences between fluorophores. Hot start touchdown PCR was performed on an Eppendorf Mastercycler (Hamburg, Germany) for 36 cycles (94°C for 2 min to activate polymerase; 16 cycles of 94°C for 30 s, 65°C for 30 s with a 0.5°C decrease in temperature for each cycle, 72°C for 30 s; 20 cycles of 94°C for 30 s, 57°C for 30 s, 72°C for 30 s; a final elongation step at 72°C for 5 min). Lengths of PCR products were determined by capillary gel electrophoresis on a 3730 DNA analyzer (Applied Biosystems, Foster City, California) at the Plant Microbe Genomics Facility, Ohio State University. Genemarker 4.0 (Softgenetics, State College, PA) was used to assign alleles by the Plant Microbe Genomics Facility. PCR products that differed by ≤ 1 bp were scored as identical in length. Lengths of the PCR products were determined by the Plant Microbe Genomics Facility and verified by the authors through direct examination of the output from the DNA analyzer.

**Table 2:** Microsatellite primers used to characterize isolates of *Sclerotinia sclerotiorum* from multiple crops in the North Central United States.

Locus^a^	Primer sequences (5’– 3’)	Repeat motif^b^	Fluorophore on universal primer^c^	Size range of PCR product (bp)	# of alleles observed
5–2	GTAACACCGAAATGACGGCCAGTCGGGCGTCATCAGATCACATGTTTATCC CTGGC	(GT)_8_	VIC	332–334	2
7–2	CAGTCGGGCGTCATCATTTGCGTATTATGGTGGGCATGGCGCAACTCTCAATAGG	(GA)_14_	PET	176–188	3
7–3	CCTGATATCGTTGAGGTCGCAGTCGGGCGTCATCATTTCCCCTCACTTGCTCC	(GT)_10_	PET	220–225	3
8–3	CACTCGCTTCTCCATCTCCCAGTCGGGCGTCATCAGCTTGATTAGTTGGTTGGCA	(CA)_12_	VIC	262–272	4
12–2	CAGTCGGGCGTCATCACGATAATTTCCCCTCACTTGCGGAAGTCCTGATATCGTTGAGG	(CA)_9_	PET	232–238	3
13–2	TCTACCCAAGCTTCAGTATTCCCAGTCGGGCGTCATCAGAACTGGTTAATTGTCTCGG	(GTGGT)_6_	VIC	311–374	7
17–3	CAGTCGGGCGTCATCATCATAGTGAGTGCATGATGCCCAGGGATGACTTTGGGAATGG	(TTA)_9_	6FAM	353–378	8
23–4	CTTCTAGAGGACTTGGTTTTGGCAGTCGGGCGTCATCACGGAGGTCATTGGGAGTACG	(TG)_10_	6FAM	405–407	2
55–4	CAGTCGGGCGTCATCAGTTTTCGGTTGTGTGCTGGGCTCGTTCAAGCTCAGCAAG	(TACA)_10_	PET	173–240	10
92–4	TCGCCTCAGAAGAATGTGCCAGTCGGGCGTCATCAGCGGGTTACAAGGAGATGG	(CT)_12_	VIC	388–395	4
106–4	TGCATCTCGATGCTTGAATCCAGTCGGGCGTCATCACCTGCAGGGAGAAACATCAC	(CATA)_25_	6FAM	535–605	20
110–4	ATCCCTAACATCCCTAACGCCAGTCGGGCGTCATCAGGAGAATTGAAGAATTGAATGC	(TATG)_9_	6FAM	382–397	6

^a^Locus names correspond to those used by Sirjusingh and Kohn [20].

^b^ The repeat motif according to Sirjusingh and Kohn [20].

^c^M13 tailed primers were used to facilitate microsatellite analysis [21].

### Analysis

Standard estimates of population genetic diversity were calculated with allelic frequencies per locus per host population using the software Arlequin v. 3.1 and Genodive v. 2.0 [22, 23, 24]. We calculated the mean number of alleles per locus and the number of private alleles (alleles unique to isolates from a particular host) for *S*. *sclerotiorum* from each of four hosts (canola, dry bean, soybean and sunflower) [25]. Because the sample sizes varied among hosts we calculated effective allele numbers and Nei’s estimate of genetic diversity adjusted for sample size by first calculating clonal diversity with Genodive and then using a jackknife approach to estimate the relationship between sample size and diversity (22). In all analyses the variance in diversity estimates decreased with increasing population size and levelled off below the actual population size sampled (S1 Fig). We also calculated pairwise *R*_st_ values between samples on the four hosts to examine differentiation between *S*. *sclerotiorum* populations from different hosts. Arlequin was also used to conduct an analysis of molecular variance (AMOVA) in order to partition the degree of genetic variability into among- and within-population components, in which a population of *S*. *sclerotiorum* was considered to be all the isolates from a given crop. Isolates from the remaining hosts were not included in the analysis, because there were insufficient isolates (1–2 per host species).

In order to assess the presence or absence of linkage disequilibrium in the four host populations we calculated an index of association (I_A_) across loci for each of the populations. The index of association developed by Brown, Feldman and Nevo [26] tests the null hypothesis that alleles at multiple loci are not linked to one another. In addition we calculated the index ṝ_d_ which accounts for the number of loci and is thus less biased. Both of these indices give an indication of the extent to which populations of haploid fungi reproduce clonally which leads to linkages among alleles at different loci rather than recombination. The indices were calculated for the full data set and for a clonally-corrected data set in which clonal genotypes were removed if they were exact repeats within the population. We implemented the analysis using the R package “poppr” developed by Kamvar, Tabima and Grunwald [27] based on work by Agapow and Burt [28].

A Mantel test was conducted using the program GenAlEx 6.5 [29] to compare genetic and geographical distance matrices and test for isolation by distance among the isolates. In order to visually assess the clustering of isolates by host we conducted a principle component analysis (PCA) on haplotype frequencies using GenAlEx. Eigen values from the first two principle components were used to calculate mean and standard error values for isolates from each host and plotted against one another.

## Results

### Isolate collection

The collection of *S*. *sclerotiorum* consisted of 145 isolates, with 136 from 12 north central states and nine from the adjacent states of Montana, Wyoming, Colorado and Manitoba, Canada (Table 1). These isolates were from nine different plant species, but 139 of the isolates were collected from four principal crops, soybean, dry bean, canola and sunflower. The majority of the isolates from dry bean, canola and sunflower were from North Dakota and Minnesota, since that is the principal production area in the United States for these crops.

### Genetic diversity

**Fig 2:**
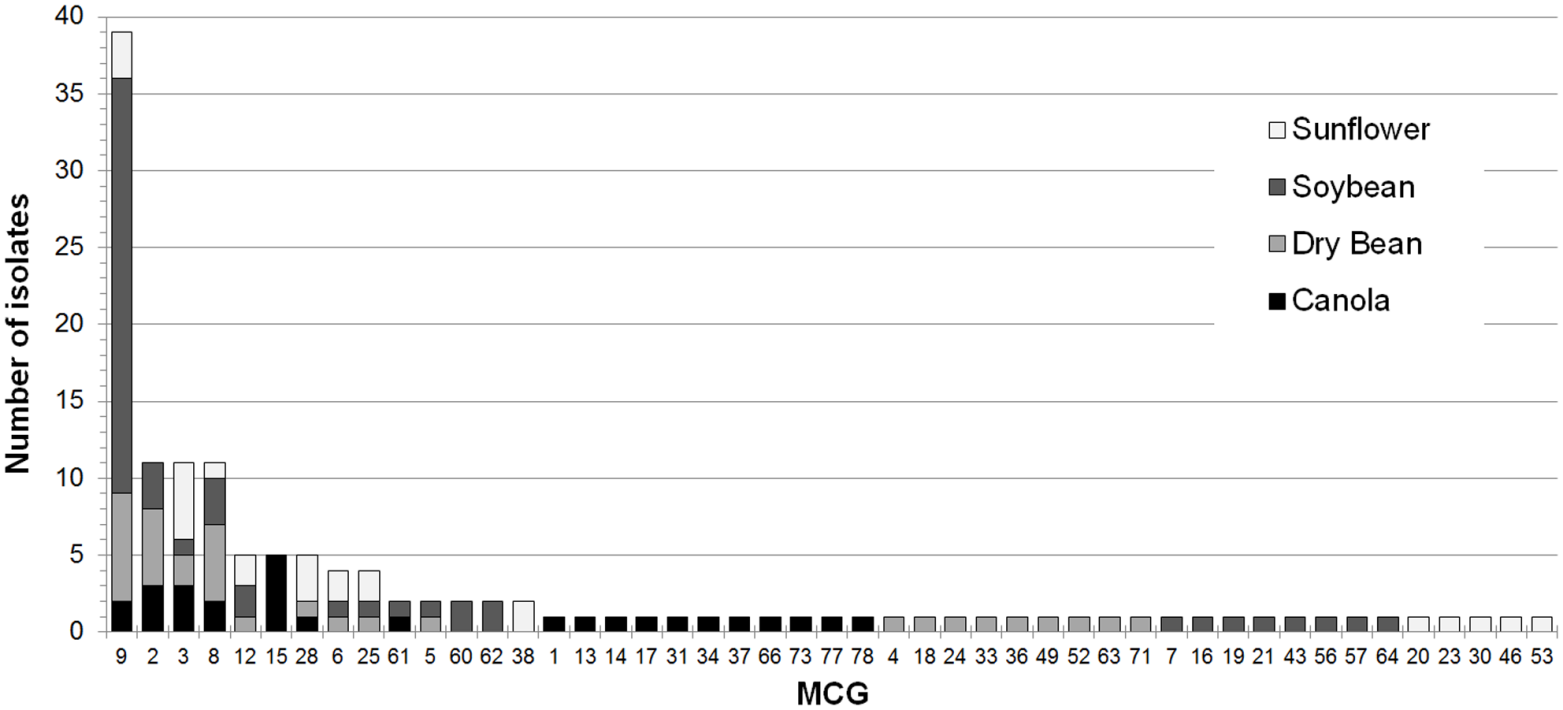
Frequency of each mycelial compatibility group (MCG) within four crops for isolates of *Sclerotinia sclerotiorum*. Distribution is almost identical for microsatellite haplotypes.

**Fig 3:**
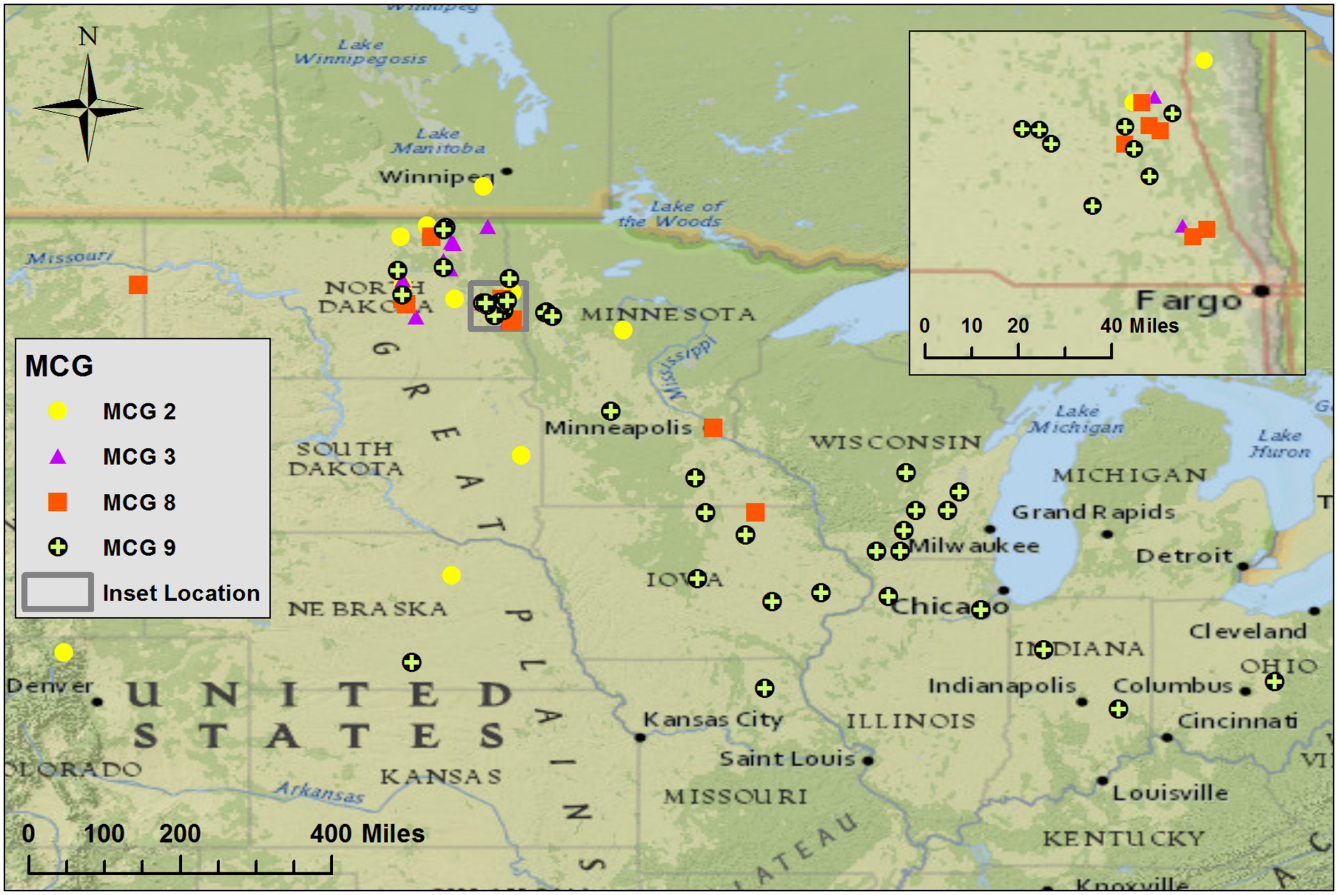
Geographic distribution of four mycelial compatibility groups (MCG) from the North Central United States. The MCG were collected from the north central area and adjacent western states and Canada. MCG 9 was the most widely distributed MCG. The map was produced with Ersi software ArcGIS 10.2 using a National Geographic basemap of North America.

Compatibility tests indicated that there were 49 different MCG’s represented by the 145 isolates analyzed in this study (Table 1). The majority of MCGs (*n* = 34) included only one isolate (Fig 2). The frequency distribution of MCGs was heavily skewed; most groups were rare, and there were only four MCGs (MCG 2, 3, 8 and 9) with ten or more isolates. The geographic distribution of isolates from the four most frequent MCGs is shown in Fig 3. The most common was MCG 9, with 41 isolates, the majority of which were collected from soybean (Fig 2). In addition, we identified 52 unique microsatellite haplotypes in the same isolates (Table 3). Haplotypes were numbered the same as MCGs, and different haplotypes within an MCG were given small letter designations after the number (e.g. 3a and 3b; Table 1). There was an association between MCG and haplotype such that isolates belonging to the same MCG either shared identical haplotypes or differed at no more than two of the 12 polymorphic loci. There were seven MCGs that had more than one haplotype (MCG 2, 3, 6, 8, 12, 25 and 28) out of 15 MCGs with more than one isolate (Fig 2; Table 1). All isolates in MCG 9 belonged to a single microsatellite haplotype. There were 11 MCGs that shared haplotypes: MCG 2 and 5, 12 and 49, 23 and 52, 12 and 56, 24 and 63, and 19 and 78.

**Table 3:** Microsatellite haplotypes of a population of *Sclerotinia sclerotiorum* from the North Central United States.

	Microsatellite locus											
Haplotype^a^	7–2	8–3	110–4	55–4	13–2	23–4	7–3	5–2	17–3	12–2	92–4	106–4
1	188^b^	268	382	189	311	407	225	332	359	238	388	572
2a	188	270	397	189	322	407	220	332	359	232	388	572D^c^
2b	188	270	397	189	322	407	220	332	359	232	388	576D
3a	186	268	390D	189	332	407	225	332	353	238	388	577
3b	186	268	394D	189	332	407	225	332	353	238	388	577
4	186	268	382	189	311	407	222	332	353	234	388	572
6a	186	268	397	189	332	407	222	332	353	234	388	568D
6b	186	268	397	189	332	407	222	332	353	234	388	593D
7	188	270	390	189	322	407	225	332	353	238	390	577
8a	188	270	390	189	322	407	220	332	359D	232	390	581D
8b	188	270	390	189	322	407	220	332	359D	232	390	585D
8c	188	270	390	189	322	407	220	332	365D	232	390	605D
9	188	268	390	189	332	407	225	334	359	238	391	568
11	188	268	393	189	332	407	220	332	359	232	391	554
12a	176	262	386	205	332	405	220	332	365	232	388	574D
12b	176	262	386	205	332	405	220	332	365	232	388	578D
13	188	272	382	193	322	407	222	332	359	234	388	565
14	188	272	393	189	332	407	225	332	361	238	390	550
15	186	272	382	173	322	407	222	332	359	234	388	554
16	186	270	397	189	332	407	222	332	353	234	388	585
17	188	270	397	189	332	407	222	332	353	234	388	574
18	186	268	382	240	332	407	225	332	359	238	388	585
19	186	270	397	181	332	407	220	334	359	232	391	550
20	188	270	382	181	322	407	222	332	353	234	391	543
21	186	270	397	189	322	407	220	332	359	232	388	572
23	186	268	397	173	311	405	220	332	359	232	388	589
24	188	270	397	189	322	407	220	332	359	232	388	572
25a	176	262	386	232	332	405	220	332	375	232	388	598D
25b	176	262	386	232	332	405	220	332	375	232	388	589D
28a	186	270	390	189	338D	407	225	332	359	238	388	572
28b	186	270	390	189	342D	407	225	332	359	238	388	572
30	186	270	382	181	322	407	225	332	353	238	388	562
31	188	268	390	181	322	407	225	334	353	238	388	572
33	188	270	390	193	322	407	220	332	359	232	390	585
34	186	268	397	177	347	407	225	332	359	238	388	554
36	186	270	394	193	322	407	222	332	353	234	388	558
37	188	268	382	193	311	407	225	332	361	238	390	572
38	186	268	382	181	311	407	222	332	359	234	390	581
43	186	270	390	185	332	407	222	332	353	234	391	585
46	188	268	382	193	322	407	225	332	359	238	391	546
53	188	268	397	181	374	407	225	332	359	238	388	572
57	176	262	386	185	332	405	220	332	367	232	388	535
60	188	268	397	181	332	407	220	334	359	232	391	568
61	188	268	397	181	332	407	225	332	359	238	388	554
62	186	268	382	173	311	405	220	332	371	232	390	577
64	186	270	397	185	332	407	222	332	353	234	388	562
66	188	270	390	189	332	407	220	332	353	232	390	581
69	188	270	382	189	311	407	225	332	378	238	395	574
71	176	262	386	228	332	405	220	332	367	232	388	535
73	186	268	382	181	311	407	220	332	353	232	390	581
77	188	268	397	181	322	407	222	332	353	234	388	565

^a^ Haplotypes were numbered the same as mycelial compatibility groups (MCGs) and different haplotypes within an MCG were given letter designations after the number.

^b^ PCR product lengths in base pairs.

^c^ D indicates where differences exist between haplotypes within an MCG.

The estimate of Nei’s genetic diversity was lowest for isolates from soybean and consistently higher for isolates from the other three hosts (Table 4). Similarly, the effective number of genotypes was lowest for isolates collected from soybean while isolates collected from canola and sunflower exhibited the highest effective number of genotypes. The number of private (unique) alleles varied between four and six across all four host populations. Because there were only six isolates from the five other plant species represented in the isolate collection, no meaningful analysis could be conducted for diversity of isolates from those hosts.

**Table 4:** Genetic diversity of isolates of *Sclerotinia sclerotiorum* on four host crops from the North Central United States.

Host	# isolates	# MCGs^a^	# haplotypes	Eff. Num genotypes^b^	*Nei’s genetic diversity* ^*c*^	# private alleles^d^
Canola	28	18	18	11.6	0.949	6
Dry bean	33	15	19	8.9	0.914	4
Soybean	53	17	20	3.6	0.738	5
Sunflower	25	13	16	12.2	0.957	4
Average		15.8	18.2	9.1	0.890	4.8

^a^ MCGs = mycelial compatibility groups

^b^ Effective number of genotypes as calculated from indices of clonal diversity in Genodive

^c^ *Nei’s* genetic diversity corrected for sample size.

^d^ Private alleles = number of alleles only found in single host.

The estimates of covariation in alleles provided by “poppr” analysis indicated that there were patterns of association among alleles in all host populations of *S*. *sclerotiorum*. Both ṝ_d_ and I_A,_ were significant for all four hosts when not clonally corrected (Table 5), supporting the hypothesis that alleles are effectively linked across loci by clonal reproduction. However, for two of the four hosts, canola and sunflower, ṝ_d_ and I_A_ were not significant when analyzed with clonal correction, a finding consistent with sexual reproduction in canola and sunflower host populations.

**Table 5:** Indices of covariance among alleles and measures of linkage disequilibrium.

	Canola	Soybean	Dry Bean	Sunflower
Sample no (n)	27	53	34	25
Clonally corrected (n)	16	11	14	5
ṝ_d_	0.11(*P*≤0.001*)	0.42 (*P*≤0.001*)	0.21 (*P*≤0.001*)	0.18 (*P*≤0.001*)
ṝ_d_, clonally corrected	0.02 (*P* = 0.09)	0.11 (*P*≤0.001*)	0.16 (*P*≤0.001*)	-0.02 (*P* = 0.66)
I _A_	1.13 (*P*≤0.001*)	4.56 (*P*≤0.001*)	2.37 (*P*≤0.001*)	1.95 (*P*≤0.001*)
I _A_, clonally corrected	0.18 (*P* = 0.09)	1.13 (*P*≤0.001*)	1.59 (*P*≤0.001*)	-0.18 (*P* = 0.66)

* Significant at the 5% level. Observed values were compared with the results of 999 randomizations of the loci and an associated *P* value is given.

**Fig 4:**
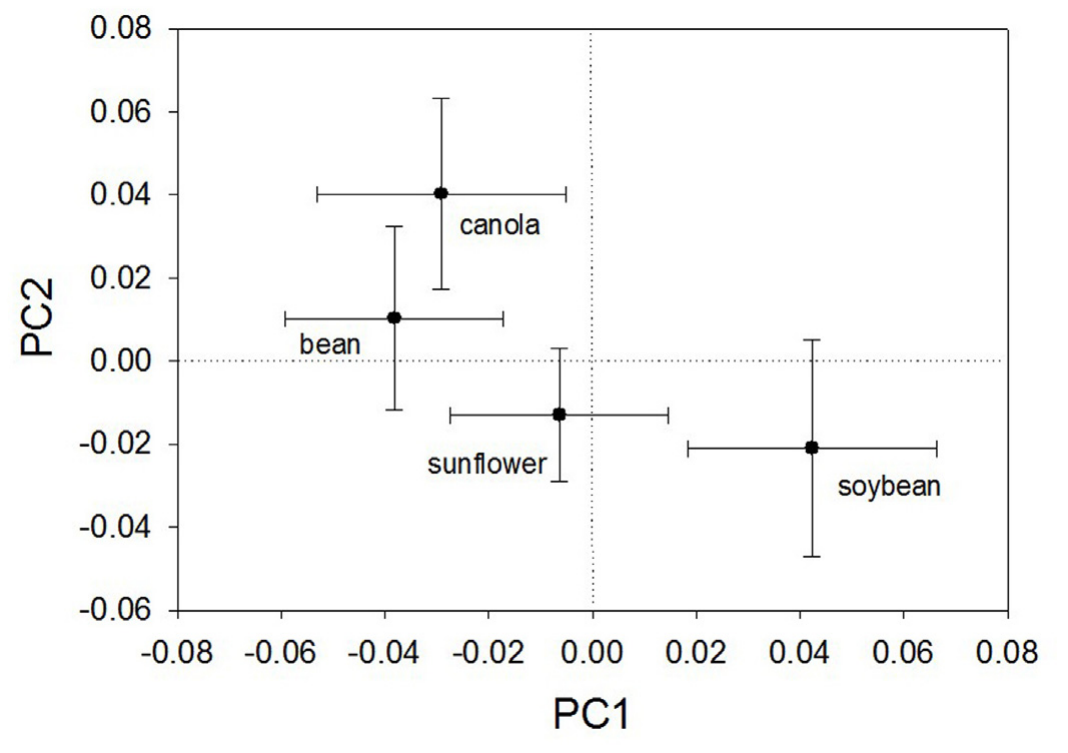
Principal component analysis of haplotype of *Sclerotinia sclerotiorum* within four crops. Plot of the mean first and second principle component. Centroids and standard error estimates are indicated.

There was little evidence for genetic isolation of *S*. *sclerotiorum* isolates among the four crops by analysis of molecular variance. Genetic variance among isolates within each host crop greatly outweighed the variance among host crops (Table 6). Ninety-three percent of the genetic variation observed in this isolate collection was explained by within-host differences in the model, indicating little divergence among the hosts. Pairwise *Rst* values for isolates between crops were relatively low (Table 7). However, the isolates from soybean consistently diverged from isolates from canola and dry bean. In addition, isolates from canola diverged from those of the other three hosts in pairwise comparisons. Principle component analysis (Fig 4) indicated isolates from canola diverged genetically from those on the other three hosts along the PC2 axis. The Mantel test for isolation by distance (99 permutations) indicated a shallow but significant slope for genetic distance as a function of geographic distance (*Y* = 1E^-07^ *X* + 11.225, *R* = 0.1, *P* = 0.01).

**Table 6:** Results of analysis of molecular variance for microsatellite haplotypes at 12 polymorphic loci for isolates of *Sclerotinia sclerotiorum* (n = 139) collected on four crops in the North Central United States^a^.

Source of variation	*DF*	*SS*	Variance component	Percent variation
Among hosts	3	35.4	0.25	7.103
Within host	135	446.3	3.30	92.897
Total	138	481.6	3.56	

^a^ Fixation Index (*F*st) = 0.071, p = 1.0 based on 1023 permutations.

The four host crops were canola, dry bean, soybean and sunflower

**Table 7:** Pairwise comparisons of fixation index values (*R*_*st*_) between isolates of *Sclerotinia sclerotiorum* from different hosts.

Host	Canola	Dry Bean	Soybean	Sunflower
Canola	–	0.147^a^ *	0.076*	0.110*
Dry Bean	<0.05^b^	–	0.048*	0.001
Soybean	<0.05	<0.05	–	0.015
Sunflower	<0.05	>0.05	>0.05	–

^a^ *R*st values are above the dash marks.

*An asterisk indicates a statistically significant (*P*≤ 0.05) divergence between host populations.

^b^ *P* values associated with *Rst* values are shown below the dash marks

## Discussion

Numerous studies have examined genetic diversity of *S*. *sclerotiorum*, but most have reported data about the pathogen collected from only one or two crops [3, 5, 6, 7, 13, 14, 15, 17, 23, 30, 31, 32, 33, 34]. Few studies have examined genetic diversity of a robust population of *S*. *sclerotiorum* across multiple crops over a large geographic area. Studies of this nature have been conducted recently in the U.S., U.K., Iran, Brazil and India [9, 10, 13, 18, 35, 36]. The most extensive study of the genetic diversity of *S*. *sclerotiorum* was conducted by Clarkson et al. [13] in the United Kingdom. They examined 384 isolates from England and Wales and found 228 microsatellite haplotypes from 12 populations collected from six crops. They reported that *S*. *sclerotiorum* had multiclonal populations in the UK. One microsatellite haplotype was widely distributed across hosts and areas.

Studies on genetic diversity in the U.S. have focused on diversity of the pathogen on individual crops [3, 7, 34, 37, 38] or diversity in a circumscribed geographic area [6, 11, 15]. There have been only two studies in the U. S. which examined genetic diversity over multiple crops collected from a large geographic part of the country. Carbone and Kohn [12] in their study on evolutionary history of haplotypes of *S*. *sclerotiorum* examined 178 isolates from seven hosts from eight states in the eastern half of the U. S. They found 34 haplotypes in that population and multiple haplotypes were recovered from some crops. Attanayake et al. [10] examined 238 isolates from four crops collected from North Dakota, Oregon and Washington. They found a high number of MCG’s and haplotypes in each population sampled.

Past studies on genetic diversity of *S*. *sclerotiorum* in the U.S. have used a variety of methods to characterize diversity, therefore comparing results between different studies, especially for haplotypes, to obtain a more complete overview of the population structure in the U.S. is rarely possible. This is especially true if one wishes to know the geographic distribution of haplotypes and which are most dominant in the population. For example, Cubeta et al. [3] used southern hybridization of BAMH1-digested genomic DNA, Carbone and Kohn [12] used the intergenic spacer region of the nuclear ribosomal RNA gene and portions of several other genes to characterize haplotypes, and Malvarez et al. [15] used the methods of both previous studies. Atallah et al. [7] and Attanayake et al. [11] on the other hand used the microsatellites developed by Sirjusingh and Kohn [20] to characterize haplotypes, with varying numbers of loci analyzed. They used 11 and nine polymorphic microsatellite loci, respectively, while the present study used 12. Furthermore, published studies using microsatellites rarely provide the PCR product lengths of each microsatellite for each isolate, hampering comparison of haplotypes across studies.

It is also difficult to compare genetic variability of *S*. *sclerotiorum* at global scales, since various methods of characterizing haplotypes have been implemented [13, 14, 30, 31, 32, 36]. Although a number of recent studies from other countries also used microsatellites, they used fewer microsatellite loci to determine haplotypes [9, 13, 17, 23, 33, 35]. Unfortunately, due to the differences in methods for characterizing genetic diversity, results from this study cannot be compared to results from several studies conducted in Canada on similar crops [5, 12, 14]. Because the North Central region borders areas included in those Canadian studies, it is highly probable that haplotypes are shared between the two countries.

The patterns of microsatellite marker allele frequencies within our study population strongly suggest that specific haplotypes of *S*. *sclerotiorum* are found on multiple crops, movement of individual haplotypes among crops occurs and that host identity is not a barrier to gene flow for this pathogen [39]. There was little evidence for genetic structure associated with host type. The absence of extensive spatial separation of centroids in the principle component analysis (Fig 4) is consistent with gene flow in the pathogen among the four hosts crops tested. Only seven percent of the overall variation in allele frequencies was due to differences among isolates on different hosts. While some haplotypes were only detected on a single crop or single sampling location, this may reflect the overall rarity of the haplotype rather than restriction to a particular crop or location. Frequently detected haplotypes were found on multiple hosts. Similar results were reported by other researchers when examining a population of *S*. *sclerotiorum* across more than one crop [13, 18] (also see review in [15]).

The analysis of linkage disequilibrium indicated a strong prevalence of clonal reproduction in the population of *S*. *sclerotiorum* from the North Central region, especially for isolates from soybean and dry bean. However, when the isolates from canola and sunflower were analyzed using a correction for clonality, there was evidence of genetic recombination within the isolates from these crops. The reasons for the differences among isolates from these host crops are not clearly understood. The extent of genetic recombination within isolates from the North Central region is being examined in greater detail in a companion study by sequencing part of the genome and analyzing allele distribution [40].

While haplotypes were generally not associated with specific crop hosts, genetic diversity in *S*. *sclerotiorum* did vary across hosts. Nei’s index of genetic diversity was highest for isolates from canola and sunflower and lowest for those from soybean. We expected to observe the greatest diversity in soybean, since isolates from this crop were obtained throughout the North Central region while canola and sunflower are primarily grown in North Dakota and northern Minnesota and more isolates were obtained from soybean than other crops. However, isolates from soybean had the lowest relative measures of genetic diversity (Table 4). The relatively low diversity on soybean is at least partially due to the fact that a large proportion of the isolates from soybean were represented by a single haplotype, as observed in other studies on the genetic diversity of this pathogen on crops [5, 13, 14].

The higher genetic diversity found within isolates from sunflower and canola may reflect an increased incidence of genetic recombination in isolates from these crops. However, other factors may also play a role in differences in genetic diversity of isolates found on different crops. Isolates from sunflower and canola were primarily collected from North Dakota and Minnesota where disease development and epidemics caused by *S*. *sclerotiorum* are common due to a favorable environment of cool temperatures combined with long wet periods. In contrast, soybean production is over a large area of the North Central region including more southerly latitudes in which disease development and epidemics in soybean are more sporadic most likely due to less favorable climatic conditions during the growing season. In North Dakota and northern Minnesota, there may be greater reproduction by the pathogen overall, both asexual and sexual, thus more chances that genetic recombination could occur.

The only evidence for genetic divergence of *S*. *sclerotiorum* was over long distances similar to those reported by Attanayake et al. [37] in their studies on canola. The pairwise genetic divergence was greatest for those isolates that were collected far from each other and was independent of host. This study suggests that isolates of this pathogen can be dispersed over large distances. Haplotype 9 was found in 10 states from North Dakota to Ohio, a distance between isolates of 2,253 km. This could be due to a genotype that is more fit than others and thus has spread and reproduced over a large area, or possibly it is due to the widespread movement of sclerotia with soybean seed in the North Central region. Of the 41 isolates of haplotype 9, 27 were collected from soybean. Isolates of haplotype 2 were also found over a distance of 1,600 km. The results of this study are similar to other studies in North America in which a general pattern of local movement was found for most haplotypes from various crops, but specific haplotypes were shown to be dispersed over long distances [3, 5, 14, 41].

In general, isolates within an MCG had the same haplotype, but 14% of the MCGs contained isolates with different haplotypes. Other studies have found similar results [3, 5, 11, 13, 14, 17]. As suggested by Hambleton et al. [14], the presence of multiple microsatellite haplotypes within MCGs may indicate that new genotypes are evolving in the population through mutation or genetic recombination. Of further interest was that 22% of the MCGs shared haplotypes, a finding also observed by several other studies [3, 11, 13, 35]. Mycelial compatibility in *S*. *sclerotiorum* is not fully understood, but it is not always associated with specific DNA fingerprints [7, 15]. The absence of linkage between MCG and microsatellite haplotype can result from sexual reproduction.

Pairwise comparisons of host populations suggest that gene flow among isolates is more common between some pairs of crops in comparison to others (Table 7). There was significant divergence in allele frequencies between the isolates from soybean and those from canola and dry bean. This could be explained at least in part by the prevalence of haplotype 9 on soybean relative to its frequency on other crops. In addition, in contrast to most of the corn-soybean production area in the North Central region where soybean is the principal host of *S*. *sclerotiorum* and other susceptible crops are rare, canola, dry bean and sunflower are major crops produced in northwestern Minnesota and North Dakota, thus gene flow between susceptible crops in this area would be more common.

An understanding of the genetic diversity and population structure of a plant pathogen is essential to designing and implementing effective management strategies. Evidence for differences in the frequency of genetic recombination across crops suggests that the response of this pathogen to management may depend to some extent on the combination of crops grown regionally. While this study found evidence for barriers to gene flow only among the most geographically distant isolates, further work with additional isolates is needed to confirm how commonly a few clones are found over considerable geographic distances.

In conclusion, this study documented a genetically diverse population of *S*. *sclerotiorum* on crops in the north central United States with little evidence of genetic structure associated with host type within the four most prevalent susceptible crops in the North Central United States. Haplotypes of this pathogen occur across crops, with certain haplotypes exhibiting a high frequency in the population. Twenty-two percent of the MCGs shared haplotypes and half of the MCGs with more than one isolate consisted of two to three haplotypes. Genetic diversity among isolates was lowest for isolates from soybean and higher for isolates from sunflower, canola and dry bean. There was weak evidence of genetic divergence over long distances. Reproduction among isolates was primarily clonal, but there was evidence of genetic recombination in isolates from canola and sunflower. The genetic diversity of this collection of isolates is currently being further characterized using high density genotyping [40]. Variation in aggressiveness of the different isolates is also being compared on multiple crops, as well as other aspects of their biology [42].

## Supporting Information

S1 Fig Variance in estimates of diversity from Genodive jackknife analysis with increasing population size. Variance is calculated as the absolute value of the difference in diversity estimate for a given population size and the overall grand mean of diversity for that host species.
(TIF)

## References

[1] Bolton MD, Thomma BPHJ, Nelson BD (2006) *Sclerotinia sclerotiorum* (lib.) de Bary: biology and molecular traits of a cosmopolitan pathogen. Mol Plant Pathol 7:1–16. 10.1111/j.1364-3703.2005.00316.x 20507424

[2] Boland GJ, Hall R (1994) Index of plant hosts of *Sclerotinia sclerotiorum* . Can. J. Plant Path. 16:94–108.

[3] Cubeta MA, Cody BR, Kohli Y, Kohn LM (1997) Clonality in *Sclerotinia sclerotiorum* on infected cabbage in eastern North Carolina. Phytopathology 87:1000–1004. 10.1094/PHYTO.1997.87.10.1000 18945032

[4] Kohli Y, Kohn LM (1996) Mitochondrial haplotypes in populations of the plant-infecting fungus *Sclerotinia sclerotiorum*: wide distribution in agriculture, local distribution in the wild. Mol Ecol 5: 773–783.

[5] Kohli Y, Morrall RAA, Anderson JB, Kohn LM (1992) Local and trans-Canadian clonal distribution of *Sclerotinia sclerotiorum* on canola. Phytopathology 82:875–880.

[6] Winton LM, Leiner RH, Krohn AL (2006) Genetic diversity of *Sclerotinia* species from Alaskan vegetable crops. Can J Plant Pathol 28:426–434.

[7] Atallah ZK, Larget B, Chen X, Johnson DA (2004) High genetic diversity, phenotypic uniformity, and evidence of outcrossing in *Sclerotinia sclerotiorum* in the Columbia basin of Washington state. Phytopathology 94:737–742. 10.1094/PHYTO.2004.94.7.737 18943906

[8] Carbone I, Anderson JB, Kohn LM (1999) Patterns of descent in clonal lineages and their multilocus fingerprints are resolved with combined gene genealogies. Evolution 53:11–21. 28565180 10.1111/j.1558-5646.1999.tb05329.x

[9] Hemmati R, Javan-Nikkhah M, Linde CC (2009) Population genetic structure of *Sclerotinia sclerotiorum* on canola in Iran. Eur J Plant Pathol 125:617–628.

[10] Attanayake RN, Tennekoon V, Johnson DA, Porter LD, del Río-Mendoza L, Jiang D, et al (2014) Inferring outcrossing in the homothallic fungus *Sclerotinia sclerotiorum* using linkage disequilibrium decay. Heredity 113: 353–363. 10.1038/hdy.2014.37 24781807 PMC4181068

[11] Attanayake RN, Porter L, Johnson DA, Chen W (2012) Genetic and phenotypic diversity and random association of DNA markers of isolates of the fungal plant pathogen *Sclerotinia sclerotiorum* from soil on a fine scale. Soil Biol Biochem 55:28–36.

[12] Carbone I, Kohn LM (2001) A microbial population-species interface: nested cladistics and coalescent inference with multilocus data. Mol Ecol 10:947–964. 11348503 10.1046/j.1365-294x.2001.01244.x

[13] Clarkson JP, Coventry E, Kitchen J, Carter HE, Whipps JM (2012) Population structure of *Sclerotinia sclerotiorum* in crop and wild hosts in UK. Plant Pathology. 10.1111/j.1365-3059.2012.02635.x

[14] Hambleton S, Walker C, Kohn LM (2002) Clonal lineages of *Sclerotinia sclerotiorum* previously known from other crops predominate in 1999–2000 samples from Ontario and Quebec soybean. Can J Plant Pathol 24:309–315.

[15] Malvarez G, Carbone I, Grunwald NJ, Subbarao KV, Schafer M, Kohn LM (2007) New populations of *Sclerotinia sclerotiorum* from lettuce in California and peas and lentils in Washington. Phytopathology 97:470–483. 10.1094/PHYTO-97-4-0470 18943288

[16] Sexton AC, Howlett BJ (2004) Microsatellite markers reveal genetic differentiation among populations of *Sclerotinia sclerotiorum* from Australian canola fields. Curr. Genet. 46: 357–365. 15549318 10.1007/s00294-004-0543-3

[17] Mert-Turk F, Ipek M, Mermer D, Nicholson P (2007) Microsatellite and morphological markers reveal genetic variation within a population of *Sclerotinia sclerotiorum* from oilseed rape in the Canakkale Province of Turkey. J Phytopathol 155:182–187.

[18] Litholdo Junior CG, Gomes EV, Lobo M, Nasser LCB, Petrofeza S (2011) Genetic diversity and mycelial compatibility groups of the plant-pathogenic fungus *Sclerotinia sclerotiorum* in Brazil. Genet Mol Res 10:868–877. 10.4238/vol10-2gmr937 21644203

[19] Schafer MR, Kohn LM (2006) An optimized method for mycelial compatibility testing in *Sclerotinia sclerotiorum* . Mycologia 98:593–597. 17139852 10.3852/mycologia.98.4.593

[20] Sirjusingh C, Kohn LM (2001) Characterization of microsatellites in the fungal plant pathogen *Sclerotinia sclerotiorum* . Mol Ecol Notes 1:267–269.

[21] Boutin-Ganache I, Raposo M, Raymond M, Deschepper CF (2001) M13-tailed primers improve the readability and usability of microsatellite analyses performed with two different allele-sizing methods. BioTechniques 31:25–28. 11464515

[22] Excoffier L, Lischer HEL (2010) Arlequin suite ver 3.5: A new series of programs to perform population genetics analyses under Linux and Windows. Mol Ecol Resour 10: 564–567. 10.1111/j.1755-0998.2010.02847.x 21565059

[23] Gomes EV, do Nascimento LB, de Freitas MA, Nasser LCB, Petrofeza S (2011) Microsatellite markers reveal genetic variation within *Sclerotinia sclerotiorum* populations in irrigated dry bean crops in Brazil. J Phytopathol 159:94–99.

[24] Meirmans PG, Van Tienderen PH (2004) Genotype and genodive: two programs for the analysis of genetic diversity of asexual organisms. Mol Ecol Notes 4: 792–794.

[25] Nei M (1987) Molecular Evolutionary Genetics. Columbia University Press, New York.

[26] Brown AHD, Feldman MW, Nevo E (1980) Multilocus structure of natural populations of *Hordeum spontaneum* . Genetics 96:523–536. 17249067 10.1093/genetics/96.2.523 PMC1214315

[27] Kamvar ZN, Tabima JF, Grünwald NJ (2014) Poppr: an R package for genetic analysis of populations with clonal, partially clonal, and/or sexual reproduction. PeerJ 2:e281 Available: 10.7717/peerj.281. 10.7717/peerj.281 24688859 PMC3961149

[28] Agapow PM, Burt A (2001) Indices of multilocus linkage disequilibrium. Mol Ecol Notes 1:101–102.

[29] Peakall R, Smouse P (2006) GENALEX 6: genetic analysis in Excel. Population genetic software for teaching and research. Mol Ecol Notes 6:288–295. 10.1093/bioinformatics/bts460 PMC3463245 22820204

[30] Carpenter MA, Frampton C, Stewart A (1999) Genetic variation in New Zealand populations of the plant pathogen *Sclerotinia sclerotiorum* . New Zeal J Crop Hort 27:13–21.

[31] Ekins MG, Hayden HL, Aitken EAB, Goulter KC (2011) Population structure of *Sclerotinia sclerotiorum* on sunflower in Australia. Australas Plant Path 40:99–108.

[32] Li ZQ, Wang YC, Chen Y, Zhang JX, Fernando WGD (2009) Genetic diversity and differentiation of *Sclerotinia sclerotiorum* populations in sunflower. Phytoparasitica 37:77–85.

[33] Sexton AC, Whitten AR, Howlett BJ (2006) Population structure of *Sclerotinia sclerotiorum* in an Australian canola field at flowering and stem-infection stages of the disease cycle. Genome 49:1408–1415. 17426756 10.1139/g06-101

[34] Wu BM, Subbarao KV (2006) Analyses of lettuce drop incidence and population structure of *Sclerotinia sclerotiorum* and *S*. *minor* . Phytopathology 96:1322–1329. 10.1094/PHYTO-96-1322 18943664

[35] Barari H, Alavi V, Badalyan SM (2012) Genetic and morphological differences among populations of *Sclerotinina sclerotiorum* by microsatellite markers, mycelial compatibility groups (MCGs) and aggressiveness in North Iran. Romanian Agricultural Research 29:323–331.

[36] Mandal AK, Dubey SC (2012) Genetic diversity analysis of *Sclerotinia sclerotiorum* causing stem rot in chickpea using RAPD, ITS-RFLP, ITS sequencing and mycelial compatibility grouping. World J Microbiol Biotechnol 28:1849–1855. 10.1007/s11274-011-0981-2 22805971

[37] Attanayake RN, Carter PA, Jiang D, del Río-Mendoza L, Chen W (2013) *Sclerotinia sclerotiorum* populations infecting canola from China and the United States are genetically and phenotypically distinct. Phytopathology 103: 750–761. 10.1094/PHYTO-07-12-0159-R 23464902

[38] Otto-Hanson L, Steadman JR, Higgins R, Eskridge KM (2011) Variation in *Sclerotinia sclerotiorum* bean isolates from multisite resistance screening locations. Plant Dis 95:1370–1377. 30731780 10.1094/PDIS-11-10-0865

[39] Hedrick PW (2011) Genetics of populations. Jones and Bartlett, Sudbury, MA.

[40] Brueggeman R, Qiu C, Nelson BD (2013) High Density Genotyping of *Sclerotinia sclerotiorum* . Phytopathology 103:S2.21

[41] Kull LS, Pedersen WL, Palmquist D, Hartman GL (2004) Mycelial compatibility grouping and aggressiveness of *Sclerotinia sclerotiorum* . Plant Dis 88:325–332. 10.1094/PDIS.2004.88.4.325 30812609

[42] Ameen G, del Rio-Mendoza L, Nelson BD (2012) Characterization of *Sclerotinia sclerotiorum* sensitivity to metaconazole in north central United States. Phytopathology 102:S4.4.

