# Supplementary figures and images for: Genetic Variation of Sclerotinia sclerotiorum from Multiple Crops in the North Central United States
Source: PLoS One. 2015 Sep 29;10(9):e0139188. doi: 10.1371/journal.pone.0139188 (PMC4587960; doi:10.1371/journal.pone.0139188)

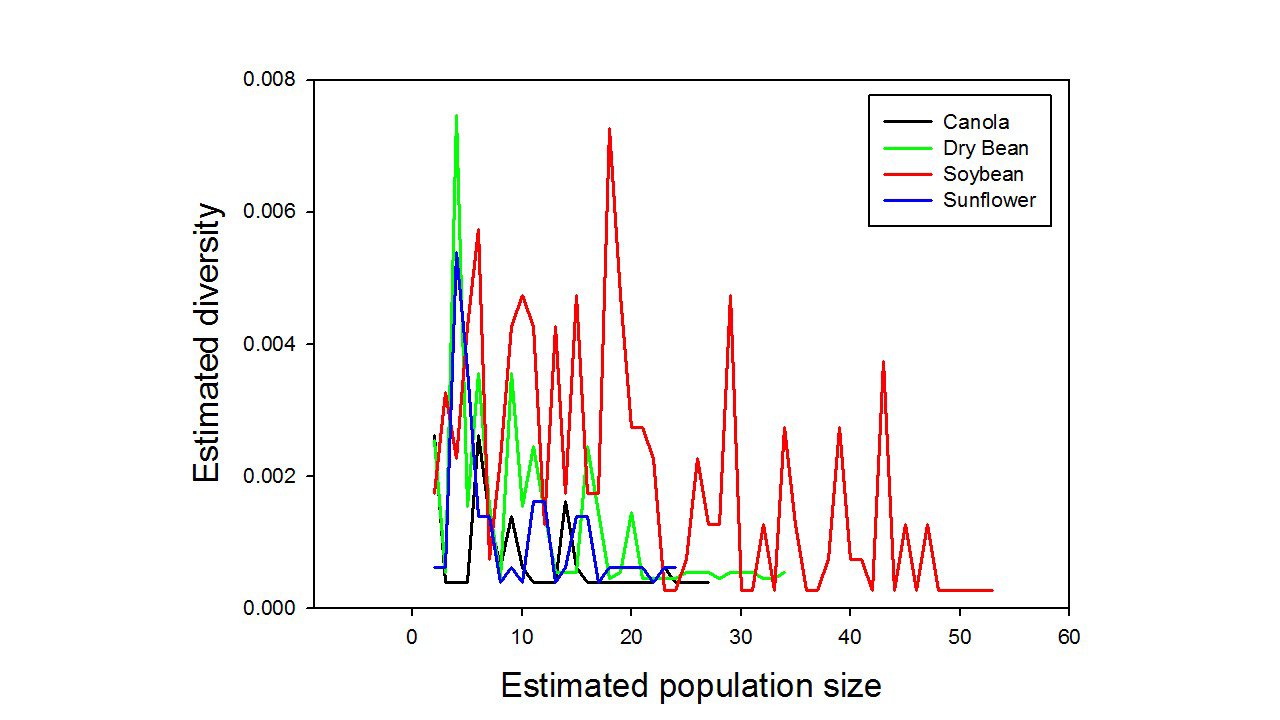

Supplement: S1 Fig — Variance is calculated as the absolute value of the difference in diversity estimate for a given population size and the overall grand mean of diversity for that host species. (TIF) [file pone.0139188.s001.tif]
